# Case

**Published:** 1860-01

**Authors:** F. Gorgas


### ARTICLE IV.

### *Case.* By DR. F. GORGAS.

ABOUT five months ago, a gentleman engaged in teaching came to my office to consult me, concerning the correction of a difficulty he labored under in articulating.

Upon examining his mouth, I found that the superior centre and lateral incisors had been separated some one or two years before by filing, for the space of one-tenth of an inch. By this operation the decay had been arrested, but at the expense of a goodly portion of each tooth filed, and the difficulty concerning which he came to ask my advice.

It at once occurred to me that the insertion of sections of teeth upon a gold plate, filling up the vacant spaces, would answer the purpose, as he was very anxious to preserve the teeth that had been filed, they being free from decay.

This was an original idea, as I do not recollect ever having heard of an artificial denture constructed in this way and for this purpose.

My first operation was to file the approximate surfaces of the teeth, so as to make the spaces between parallel. After this was done, the teeth not being as sensitive as I imagined they would from so much filing, I took an impression of the mouth in gutta-percha, and swaged up a gold plate, allowing it to extend between the filed spaces in the same manner it should do over the alveolar ridges, where an entire tooth was to be inserted.

My next operation was to carve sections of teeth suitable for the purpose, and to insert in them small pieces of platina to answer the place of rivets, bent so as to hold firmly in the teeth. Upon these platina backings I melted gold scraps.

It was impossible to use common plate teeth ground sufficiently small to enter the spaces, as it would have brought the rivets so near the ground surface as to take away the strength required. It was also necessary to have the spaces between the teeth perfectly parallel, so as to insert and remove the piece with ease.

This piece I inserted upon clasps. Some three months after this piece was made, a friend of my former patient, also engaged in teaching, and like him laboring under the same difficulty, came to me for a similar piece, that of his friend answering so well. For him I inserted two sections of teeth also, and two first bicuspids on an atmospheric pressure plate.

I used an atmospheric pressure plate in this case because all of the bicuspids and molars remaining in the mouth were filled, and consequently not fit to be clasped.

In this case the sections of teeth inserted were not more than one-twelfth of an inch in width.

This last piece has now been worn one month and a half, and as far as I know, has also given satisfaction.

